# Advertising Operations in the Daily Papers

**Published:** 1886-12

**Authors:** 


### *ADVERTISING OPERATIONS IN THE DAILY PAPERS.*

The New York *Medical Record*, commenting upon the suggestion of the committee on ethics, of the Medical Society of the County of New York, concerning repression of the growing tendency to publish surgical operations in the newspapers, says that such action is timely and should receive the unqualified support of every practitioner of medicine and surgery. "The names of every one of those who have heretofore winked at this breach of propriety and good sense are well known, and whenever the subject is brought up, they are promptly enumerated as the recognized violators of what honorable professional gentleman endeavor to sustain and maintain. These trenchers upon the good name of the profession can, with perfect safety, assure themselves that they do not occupy an enviable position." Of late years, thanks to the support which the JOURNAL has received from the better element of the profession, in our efforts to suppress the introduction of newspaper advertising, Buffalo, has been comparatively free from this evil, but it is a growing tendency and we find our editorial confreres in other cities "applying the lash" to these offenders with a good deal of vigor. Even staid and respectable old Baltimore has its surgeons who are attended by the ubiquitous and gifted reporters, who, without any medical education, is still able to give all the details of an operation in terms glowing with extravagance, if not inaccuracy. From an editorial in the *Maryland Medical Journal*, we quote as follows: "We profess to abhor and discountenance the 'red-handed quack,' but the scale by which he is measured slides more easily year by year. The aforesaid rosy individual advertises in the proper columns that he can cure any or a large number of diseases and he pays for his advertisement. In another column of the same paper we may read that 'Professor A., ably assisted by Professors B., C. and D., performed, with great skill, a very difficult operation, the only one of the kind ever done,' or the removal of an *enormous* tumor by Dr. So-and so; patient doing remarkably well." Now these gentlemen *who had nothing to do with the insertion of this item*, may read it in the morning papers with great complacency, whether they recognize or not the fact that this is the most subtle, the most skillful and often the cheapest mode of advertisement.

We think we cannot urge too earnestly upon the members of of the profession, the importance of this matter, which, while it seems of so little moment is yet capable of such great abuse. Physicians should see to it that such items as those spoken of *do not* get into the daily papers. Of course in police and accident cases the mention of the attending physician's name is often unavoidable, but surely there is no excuse for the daily notice that "Dr. —— reports his patient better." Much less offensive, but at the same time we think, open to criticism, is the advertisement of public institutions, in which the names of medical men prominently appear. It is for the benefit of such institutions that the public should be informed that they are attended by well-known and skillful physicians—but it cannot but be annoying to such men to see their names placarded upon the public highways. In connection with one of our city hospitals such an advertisement has recently appeared bearing the names of doctors who occupy a foremost rank in the profession, men, too, who have been singularly free from any suspicion of advertising tendencies. We learn that, at the request of the medical men concerned, this advertisement was promptly suppressed. It is only by thus avoiding even the "appearances of evil" that we are able to strongly and consistently condemn the advertising doctor when he appears in the ranks of the profession.

